# Performance Analysis in Olympic Sailors of the Formula Kite Class Using GPS

**DOI:** 10.3390/s21020574

**Published:** 2021-01-15

**Authors:** Israel Caraballo, José Luis González-Montesinos, Francisco Casado-Rodríguez, José V. Gutierrez-Manzanedo

**Affiliations:** 1 GALENO Research Group, Department of Physical Education, Faculty of Education Sciences, University of Cádiz, Cádiz, 11519 Puerto Real, Spain; israel.caraballo@uca.es; 2 Research Unit, Biomedical Research and Innovation Institute of Cádiz (INiBICA), 11519 Puerto Real, Spain; 3 Department of Physical Education, Faculty of Education Sciences, University of Cádiz, 11519 Puerto Real, Spain; josegu.manzanedo@uca.es; 4 Andalusian Sailing Federation (FAV), Puerto Sherry, 11500 El Puerto de Santa María, Spain; francisco.casado@fav.es

**Keywords:** performance, sailing, kitesurfing, Olympic sailors, GPS

## Abstract

Formula Kite is an Olympic sport that mainly differs from other kitesurfing modalities for the use of a hydrofoil. It is considered an extreme sport due to the great technical ability required. Regarding performance, the variables that determine performance in a real competition situation have not been studied, and even less so with Olympic sailors. Therefore, the objective of this study was to determine the technical and tactical variables that differentiate elite sailors. The sample consisted of 42 Olympic sailors of the Formula Kite class, who were evaluated in three World Cups. Using a GPS device, the speed, distance traveled, maneuvers, and time spent on the courses of upwind, downwind, and beam reach were recorded. The highest-level sailors presented a higher speed in upwind/downwind/beam reach and a shorter time in upwind and beam reach. Performance seems to be more strongly influenced by technical variables, such as speed, than by tactical variables.

## 1. Introduction

Kitesurfing is a surface water sport that combines aspects of other water sports such as sailing, surfing, windsurfing, wakeboarding, and paragliding. Since 1990, its popularity has grown exponentially, with 60,000 persons beginning to practice this sport around the world every year [1,2]. This sport requires great technical skills and is considered an extreme sport. In it, the sailor is powered by using a kite that is used as a sail and glides over water by using a board. This kite is controlled with a bar and, by means of lines of ropes, it is kept at a distance of about 25 m from the sailor. The kitesurfer can reach speeds of up to 30 knots, depending on the wind speed, the size of the kite, and the state of the water surface. In kiteboarding races, the main disciplines recognized by the International Kiteboarding Association (IKA) are the course racing, freestyle, wave, slalom, and speed [3]. The energy demand is determined by the type of discipline practiced by the sailor, and the course racing discipline can be considered as an activity of moderate intensity. It is characterized by the low increase in lactate levels and maximum oxygen consumption, but with a large increase in heart rate when the wind speed is low (12 to 15 knots) [4]. In the course racing discipline, the sailor must complete a regatta in the possible shortest time, sailing upwind and leeward in a regatta circuit bounded by buoys.

Among the different classes that belong to kitesurfing, the Formula Kite class is the only one included in the Olympic Games, and it is a high-performance hydrofoiling class that uses registered series production equipment. The equipment must comply with the measurements published on the registered series production equipment lists. The IKA Formula Kite class aims to regulate the equipment used in kiteboarding regattas. These rules are developed to allow competitors with different characteristics to compete on an equal playing field. The development of equipment within the limitation of these class rules is encouraged. This class debuted at Rio de Janeiro 2016 Olympic Games, and in the Olympic Games in Paris 2024, it will be officially an Olympic discipline. The regatta competition format will be within the course racing discipline, involving the speed and tactics. This type of regatta is the most common to hold competitions on kiteboarding boards equipped with hydrofoils [3].

The main feature that differentiates the Formula Kite class from the other classes is the use of a hydrofoil. The hydrofoil is an appendage primarily used to produce vertical lift and/or affect leeway and may include a mast, front wing, fuselage, and stabilizer, and only one foil system is permitted to be attached to the hull. Only hydrofoil parts licensed by the World Sailing by the end of 2021 may be used in international competitions from 1 January 2022 onwards. Sailors can only register a kite based on its size range, and two ranges determine the size of the kite: large (equal to or greater than 15 m^2^) and medium (between 11 and 15 m^2^). The board (hull) can have a maximum length of 1550 mm and a maximum beam of 500 mm, and the total weight of the hull, the foil system, and footstraps used during the race cannot be less than 5.5 kg [5].

As in other sports, psychology is a key performance variable in sailing sports, and decision-making is a variable that is related to sailing tactics [6]. When the sailor is sailing, he/she must carry out a decision-making process in order to interpret and respond to the stimuli of the environment, and such process is influenced by the ability to visualize [7]. This variable determines the spatial orientation of the sailor, and the greater the development of this capacity, the better the position in which he/she will be located in the starting area and the better the route chosen during the regatta will be, using the positions of buoys as a reference [8,9].

The physiological responses of dinghy sailing sports have been previously described in studies that were mainly focused on the measurement of energy metabolism, oxygen uptake, and heart rates for both on-water and simulated upwind sailing. Energy metabolism studies show values between 35% and 45% VO_2max_ [10,11,12]. Other studies have shown that the level of aerobic capacity in elite sailors is very similar to that of elite athletes in other sports disciplines [10,12]. In this sport, the demand for aerobic capacity is directly related to the wind speed. This demand increases, as the wind speed increases, with sailors showing values of 80–90% VO_2max_ when the wind speed is 20 knots [11,13]. On-water upwind sailing sailors have shown heart rate records of 132 ± 12 beats per minute with light winds (4–9 knots), 158 ± 11 beats per minute with moderate winds (10–16 knots), and 165 ± 8 beats per minute with strong winds (16–30 knots) [14].

In sailing sports, such as windsurfing, in which the sailor glides on water with a board, the energy metabolism required is mainly aerobic. The technical and tactical decisions made during the regatta require a combination with anaerobic metabolism [15]. The mean oxygen consumption can reach 80% VO_2max_, the mean maximum heart rate can be around 90%, and the blood lactate concentration can be as low as 9 mmol L^−1^ [11,16,17].

To our knowledge, only few studies have analyzed the physiological demands of kiteboarding. In this class, the mean oxygen consumption can reach 54% VO_2max_, the mean maximum heart rate can be around 80%, and the blood lactate concentration can be as low as 2 mmol L^−1^, measured on-water and with a wind speed between 11 and 15 knots, although no study has been conducted during a real competition [4,18,19].

In sailing, there is a high level of inter-individual dynamics; thus, the performance will be conditioned by the ability of the sailor to create uncertainty in other competitors [20]. To achieve this uncertainty, the sailor can modify his/her trajectory, increase the speed in the wind rolls or vary the angle of the heel. However, the studies carried out in the laboratory do not allow evaluating the effect of competitors, whereas the studies conducted in real competition situations allow collecting this interaction, including the competitors and the actions of athletes [21]. Therefore, the analysis of the athlete in a competition situation could provide a structure to analyze the athlete’s performance skills, that is, the perceptual-motor skills that are used to respond to the different situations’ limitations, to the other competitors, and to the changing characteristics of the environment [20,22].

The use of GPS devices designed specifically for applications in the sports field was consolidated in 2003, since it was from that date when the advancement of technology allowed them to withstand heat, humidity, and potential impacts produced during sports practice [23]. GPS devices provide detailed information on external loads, such as movement patterns and physical activities of the athlete during training or during competition [24]. The external load is the movement or work that an athlete performs during sports practice, and it is independent of the internal responses to a stimulus [25,26]. This information includes speed, distance traveled, and time. This type of devices is widespread in the evaluation of sports performance due to its validity and reliability when registering variables of speed, distance, maneuvers, and time [19,20,27,28,29]. Therefore, a higher level of knowledge regarding the displacement data of those who achieve better positions in the regatta could provide relevant information to better understand the technical and tactical aspects that determine performance in a regatta in kitesurfing.

In sailing sports, techniques and tactics play a relevant role, the performance of the regatta will be determined by the average speed, the velocity made good (VMG), the distance traveled, and the number of maneuvers [27,30]. The technical level of the sailor in the different courses carried out in a regatta will determine the speed of the board, and the VMG in the windward and leeward courses is considered the most important variable to evaluate the performance of the sailor [31,32]. The distance traveled is another variable that differentiates the higher- and lower-level sailors, with the higher rankings being composed of those who travel the shortest distance to complete the range established in the regatta [27,30]. The maneuvers carried out by the sailor can determine the speed of the board, since there is a loss of speed in each maneuver; therefore, they must be carried out as efficiently and quickly as possible to minimize the decrease in speed [17].

Although the literature shows the relationship between performance and technical (velocity) and tactical (distance and maneuvers) in Windsurfing [27,30] and Laser [31,32] classes, to our knowledge, no study has provided the variables that determine performance in the Formula Kite. Similarly, the importance of each of the courses that are developed during a regatta in the Formula Kite class has not been determined. Most of the studies in kitesurfing are focused on analyzing the type of injuries suffered by sailors [33,34,35,36], others have focused on analyzing physiological demands [4,37], and only one has focused on analyzing performance-related variables in a real competition situation [19]. Therefore, the objective of the present study was to identify the technical (velocity) and tactical (distance, maneuvers, and time of the legs) variables that determine sport performance in the Formula Kite class.

## 2. Materials and Methods

### 2.1. Participants

**Figure 1:**
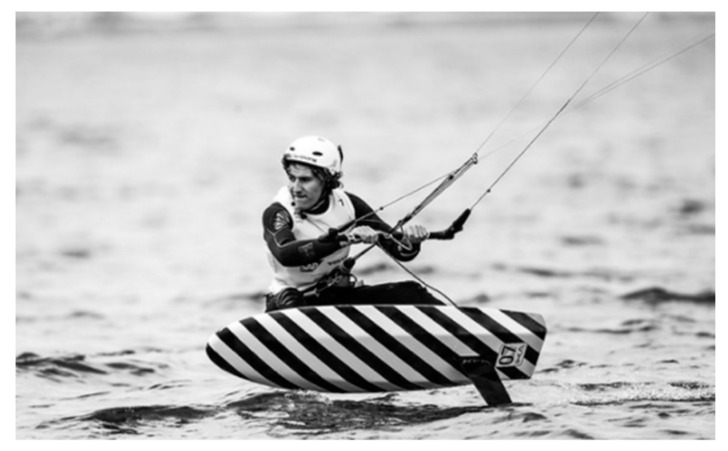
Formula Kite class sailor.

The study sample consisted of 42 Olympic sailors (7 female) in the Formula Kite class, with an age range of 15 to 49 years (Figure 1). The data were collected in the months of January and February of the year 2020 from the World-Sailing^®^ [38], which is the commercial entity of the International Sailing Federation (ISAF) that manages the competition system at a worldwide level. The total sample of 42 sailors was divided into three groups based on their performance levels: high level (T1), medium level (T2), and low level (T3). The eligibility criteria for inclusion of the sailors in the study were the following: (1) the date of birth data had to be on the World-Sailing^®^ website [38]; (2) the sailor must have completed all the races that were analyzed in the regatta. The median value in the ranking was used to divide the sample in T1 (*n* = 12; 0 females), T2 (*n* = 15; 0 females), and T3 (*n* = 15; 7 females).

### 2.2. Regattas

The analyzed regattas were three Formula Kite class World Cups: Melbourne (Australia, 2016), Santander (Spain, 2017), and Marseille (France, 2019). These three regattas are qualifying competitions for the World Cup, and they are also qualifying tests to participate in the Olympic Games. The studied variables were obtained through the SAP-Sailing^®^ application [39]. This application used a GPS device placed on the sailor. From this device, data were transmitted and processed in real time by the application, obtaining information from the sailor about velocity, distance, maneuvers, and time during the regatta. The tracker used was a small 60 g device containing GPS, mobile connection, and battery. The device sampled the position of the competitor at frequent intervals of 5 Hz and sent the data to the system via the mobile network.

**Figure 2:**
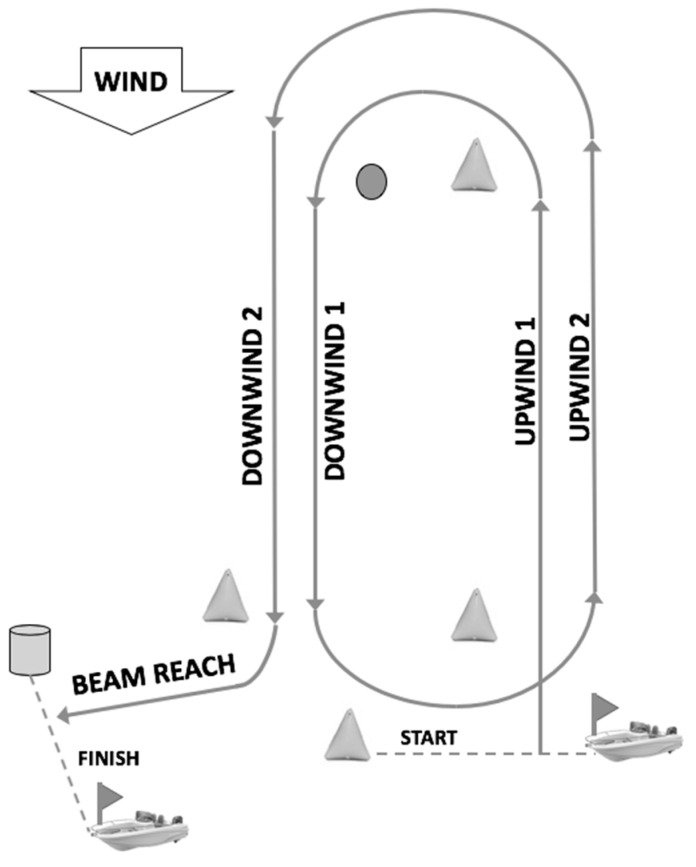
Regatta race course.

The race course consisted of 4 windward/leeward legs, and the true wind was at right angle to the sailing craft, with lengths appropriate for the condition, with 2 in windward (upwind), 2 in leeward (downwind), and 1 in beam reach (Figure 2). A total of 17 races were analyzed: 5 races in Melbourne, 6 in Santander, and 6 in Marseille.

### 2.3. VMG

**Figure 3:**
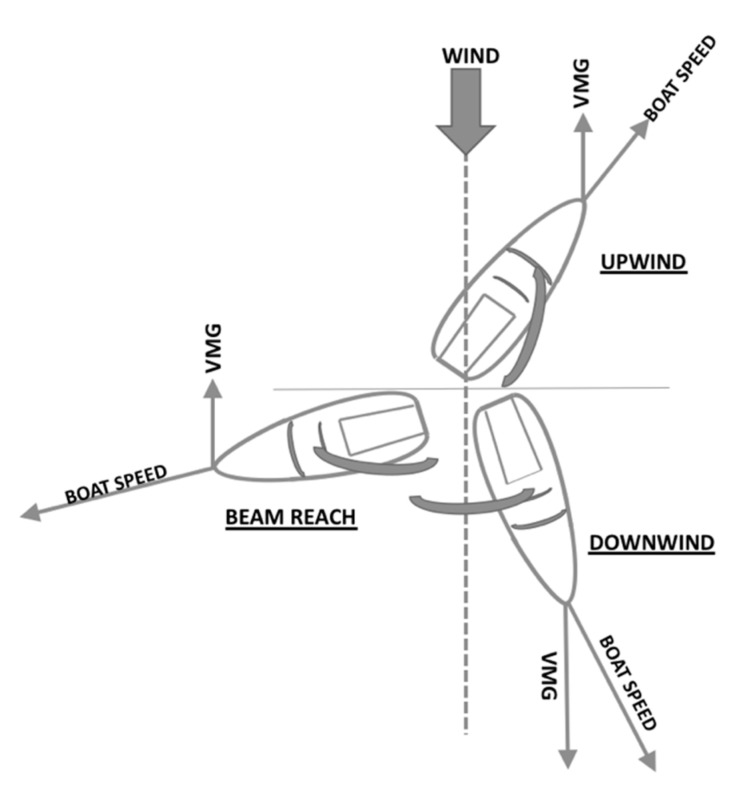
Velocity made good (VMG).

VMG determines the performance of the board according to the maximum speed it can reach as a function of the course, and it considers the vertical component of the speed with respect to the wind direction [31]. This velocity is the best combination of the speed and sailing angle, making the board advance as far as possible [40,41] (Figure 3).

### 2.4. Statistical Analyses

The data are presented as means (M) and SD. The level of significance was set at *p* < 0.05. The SPSS v20.0 software (SPSS Lead Technologies Inc., Chicago, IL, USA) was used for the statistical analyses. The normality was also verified, using the Kolmogorov–Smirnov test. The analysis of variance (ANOVA) was used to analyze between groups and to analyze possible differences attending to the performance level (T1, T2, and T3), and they were divided into three groups according to the tertiles. Tukey and Games-Howell pos hoc tests were performed, when statistically significant differences were detected. Due to the measurement levels present in the data, a nonparametric Kruskal–Wallis test was applied to establish differences between some variables. ANOVA effect size (ES) was calculated using partial eta-squared ($\eta_{p}^{2}$) with <0.25, 0.26–0.63, and >0.63 considered small, medium, and large, respectively [42].

## 3. Results

Table 1 shows the descriptive analysis in all sailors, boys, girls, and each of the groups of sailors according to their levels of performance.

**Figure 4:**
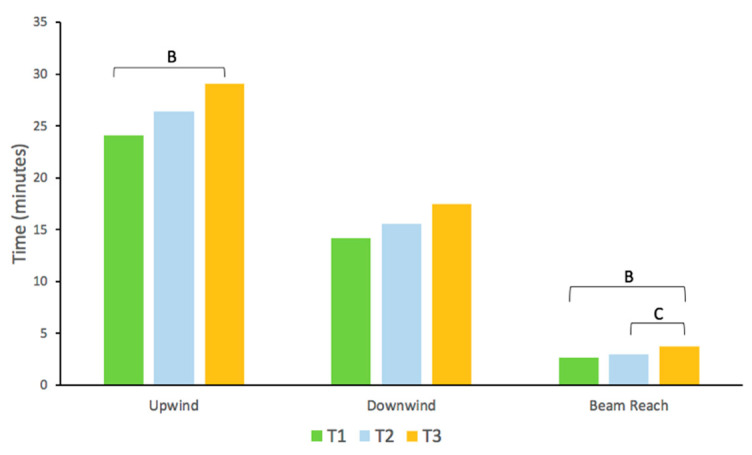
Comparison between the groups of sailors with different performance levels (T1, T2, and T3) in upwind and beam reach for time. ^B^: statistically significant difference between T1 and T3; ^C^: statistically significant difference between T2 and T3; T1: high-level sailors; T2: medium-level sailors; T3: low-level sailors.

Table 2 shows the results of the descriptive analysis for each of the groups of sailors according to their levels of performance. It was observed that the sailors of group T1 obtained a greater mean velocity and VMG compared to the sailors of group T3 in upwind, downwind, and beam reach, The mean velocity of the athletes of group T2 was greater than that of the sailors of group T3 in upwind and beam reach. Similarly, the VMG was higher in group T2 compared to in group T3 in the entire analyzed course. According to the time variable, the sailors of group T1 showed a shorter time than those of group T3 in beam reach (Figure 4). The analysis of the mean values did not show differences in distance and maneuvers in upwind, downwind, or beam reach.

Table 3 shows that the maximum mean velocity was achieved in downwind (27.9 knots) while the maximum VMG was obtained in beam reach (27.2 knots). In upwind, the lowest values of the mean velocity and VMG were 13.4 and 6.2 knots, respectively. When analyzing the distances traveled in the different courses, it was observed that the beam reach was lower in groups T1 (2.1 km), T2 (2.3 km), and T3 (2.3 km). The course with the longest distance traveled was upwind (10.3–10.8 km), and the shortest distance was traveled in the beam reach course (2.1–2.3 km). Beam reach was the course with the least number of maneuvers (0–4), and the upwind heading was the one with the most maneuvers (18–45). The greatest number of maneuvers was recorded in group T3 in downwind (42 maneuvers). Regarding time, it was observed that downwind is the course in which the sailors spent the longest time (16–55 min). In beam reach, a minimum time of two minutes was recorded.

## 4. Discussion

The aim of this study was to investigate some relevant aspects involved in the technical (velocity) and tactical (distance, maneuvers, and time) performance in the Formula Kite class course racing, by monitoring external (GPS) workload parameters during World Championship regattas in Olympic sailors. To our knowledge, this is the first study to evaluate technical and tactical variables, such as velocity, distance, maneuvers, and time, in a real regatta situation as a function of the performance of the sailors, specifically in Formula Kite class sailors. The current findings regarding velocity, distance, maneuvers, and time provide relevant and valuable information on the intensity level that may be used by elite kitesurfers for the preparation of a specific training program. However, the lack of actual velocity, distance, maneuvers, and time measurements during the on-water testing trials constitutes a major limitation of our study and makes it difficult to compare these technical and tactical variables with those reported in the literature. Given the absence of scientific data related to kitesurfing, we compared the results of our elite group of kitesurfers with those observed in highly trained windsurfers.

The results of our study showed that the highest-level sailors achieved the highest speed in the courses of upwind, downwind, and beam reach, in terms of the average speed and VMG. These results could be very interesting for coaches and sailors, because they showed that the speed of the elite sailor is not affected by a certain course. Consequently, the training sessions should focus their training on improving their speeds in each of the courses established in the regatta. Speed as a performance factor in windward and leeward courses has also been confirmed in the windsurfing class [27,30].

Analyzing the speeds in each of the courses, it was observed that the sailors were faster in the beam reach course, followed by downwind and upwind, with the latter being the course in which the lowest speed was reached. These results are very similar to those obtained in other studies in kitesurfing class sailors [4,19]. In both studies, elite kitesurfers were evaluated and, in one of them, several official regattas were analyzed. This is the first study to determine the fastest course in the Formula Kite.

Regarding distance, no differences were found between the three groups of sailors. However, other studies have shown that the sailors with a higher level completed the course with a shorter distance [30]. The sample of this study consisted of 14 elite sailors (5 women) from the windsurfing class in a real competition situation. This difference in the results could be due to the fact that the variables that determine performance may be different in each of these classes. The Formula Kite sailors could travel a greater distance in order to obtain a better angle of navigation to reach a higher speed. To our knowledge, we must emphasize that, to date, no study has analyzed the distance traveled by the sailor and how this distance is related to performance. Furthermore, our study is the only one that analyzes the three possible sailing courses during a regatta.

Based on these results, we can say that speed and performance in the Formula Kite class are not determined by the number of maneuvers carried out in the windward and leeward courses. Dividing the sailors into two homogeneous groups (median) based on the number of maneuvers they perform in each of the courses, it was observed that there were no significant differences when comparing the values of speed (mean velocity and VMG) in any of the navigation courses (results not shown). However, in the case of the windsurfing class, the number of maneuvers is a determining factor for performance [27,43]. Every maneuver reduces the speed of the board, and the highest level sailors are those who perform the least maneuvers in the upwind and downwind courses. This could indicate that the sailor should not limit the number of maneuvers during the race so that the boat maintains its maximum speed.

Although our results have not shown significant differences between the three groups in the range of distance covered and maneuvers carried out, the data reported in the present study may provide important information to prepare training programs for sailors (Table 2).

The time taken to complete the upwind and beam reach courses is a determining variable in differentiating higher-level sailors from lower-level sailors. Higher-level sailors spend less time in the upwind and beam reach courses. This variable is related to speed; thus, it is logical that the highest-level sailors also used less time when reaching a higher speed in each of the courses. We have observed that the time taken to perform some of the legs was longer than the total time recommended by the IKA to complete the entire tour, since the estimated time to complete them is 30 min [44]. Therefore, it seems that the race judges determined greater distances in accordance with the duration of the race suggested by the IKA. During kitesurfing regattas, especially in the crossing mode, the sailor makes repeated and prolonged movements on the board that are characterized by isometric efforts in the flexor-extensor musculature of the knee and elbow, and thus, the more intense the effort, the greater the time for the sailor to perform it [4]. This should be taken into account by sailors and coaches, since, as the sailing time increases, it is logical that the required physiological demands also increase. However, and in contrast to our results, other studies have found that the distance established by the race judges was adequate to complete the course in the time stipulated by the IKA [19]. It is possible that this difference is due to the meteorological characteristics that occurred in each of the regattas, and especially to the speed of the wind, since it is one of the determining factors in establishing the length of each of the legs.

The GPS information provided can be of great help to understand how competitions develop in sports that cover long distances [43]. The use of this device for the analysis of the distance traveled has been evaluated in sports such as cross-country skiing [45], orienteering races [46], mountain biking [47], surfing [48], windsurfing [27], and kitesurfing [19]. Current GPS devices usually use sampling frequencies of 1, 5, 10, 15, and 20 Hz. The results of several studies indicate that the use of frequencies from 1 Hz is suitable for recording speeds and distances and that these show great precision during basic linear movements [23,49]. A mean precision error of 5.6% is generally accepted for GPS devices in measuring motion when comparing running with walking and between linear and nonlinear movements [50]. It is considered that this error in precision is produced by the speed reached by the athlete and that such error increases with the speed reached [51]. In general, it seems that the higher the sample rate, the more valid GPS becomes for measuring distance. In kitesurfing, the movements are normally linear and continuous; in addition, the changes of direction are usually slow, and thus, the use of a recording frequency higher than 1 Hz seems to be adequate for this sport [52]. In addition, the combination of GPS with location-based services, such as the SAP-Sailing application, allows following competitions and training in real time and obtaining data on the sailor’s technique and tactics, which can be later analyzed to improve performance. Another important aspect of sports GPS devices is their size; they should not limit the movements of athletes, and their weight determines the place where the device will be located [53]. The device used in our study weighed less than 70 g, and thanks to these characteristics, the use of this type of device has become widespread in elite sports such as rowing and kayaking. Thus, we can consider that it did not interfere with the sailor’s technique or tactics [54,55]. Therefore, these devices could report information on technical and tactical aspects of the sailor that could be evaluated during training and later applied to competition.

Our study is not exempt from limitations. Firstly, regarding the number of participants analyzed, the study sample consisted of only 42 elite sailors in Formula Kite class World Championship events. However, this represents 84% of the total number of sailors who have participated in the sport events described. Secondly, no information was gathered about the height and weight of the sailors. It would have been interesting to analyze those variables; unfortunately, it was not possible to include such data in this study. The analysis and understanding of performance in complex sports, such as sport sailing, must take into account changes in wind, waves, changing tides, competitors, and boat tuning. Therefore, although the technical skills of the sailor are key to achieving the highest speed of the boat, the distance to complete the course can vary, since the wind and waves can change direction and speed along the course [6]. These unpredictable environmental factors add to the level of complexity in sport sailing [20,22]. Moreover, future studies could be focused on the analysis of the physiological demands of the kitesurfers in different wind conditions. To carry out this type of research, multiple types of sensors could be used with which it would be possible to evaluate these variables and determine possible measurement errors.

## 5. Conclusions

In conclusion, this study has shown that the Formula Kite class exhibits unique performance characteristics, even when compared to other dinghy sports included in the Olympic program. The variables related to tactics, such as the distance traveled and the maneuvers performed, are not key to differentiating the sailors based on their performance levels in the Formula Kite class. However, speed (average speed and VMG) and time spent traveling upwind and beam reach seem to be the variables that can best distinguish between a good and a bad sailor. The speed of the board is the variable that best differentiates the levels of the sailors in the Formula Kite class; therefore, this variable would be more important than tactics in the performance of the sailor. The time it takes to cover the distance on the upwind and beam reach courses is shorter for elite sailors. These results can be of great interest to coaches and physical trainers of sailors, since they can help them to better target the objectives of their training, specially specific sailing trainings focused on improving board speeds in windward–leeward courses. Lastly, GPS devices are a very useful tool for monitoring dinghy training.

## Figures and Tables

**Table 1:** Mean ± SD of the age variable in all sailors, females, males, and performance groups (T1, T2, and T3).

	All (*n* = 42)	Female (*n* = 7)	Male (*n*= 35)	T1 (*n* = 12)	T2 (*n* = 15)	T3 (*n* =15)
Age (years)	25.7 ± 8 (15–49)	33.1 ± 13.9 (15–49)	24.2 ± 5.5 (16–47)	23.3 ± 4.6 (20–37)	24.2 ± 3.8 (16–32)	29.2 ± 11.7 (15–49)

Note: (minimum–maximum).

**Table 2:** Data of mean velocity (M), VMG, distance, maneuvers, and time in upwind, downwind, and beam reach in groups of sailors with different performance levels (T1, T2, and T3).

Variables	Upwind			Levene Test		*p* Value	*d*	95% CI
	T1 (*n* = 12)	T2 (*n* = 15)	T3 (*n* =15)	F	*p* Value			
Mean velocity (knots)	19.2 ± 1.2 ^B^	18.3 ± 1.1 ^C^	16.1 ± 1.8	2.82	0.07	0.0	0.46	[17.17; 18.38]
VMG (knots)	12.2 ± 1.4 ^B^	11.2 ± 1.4 ^C^	9.3 ± 1.5	0.005	0.99	0.0	0.412	[10.3; 11.46]
Distance (km)	14.4 ± 5.4	14.9 ± 4.8	14.3 ± 4.4	1.48	0.23	0.94	0.003	[13.17; 16.14]
Maneuvers (number)	24.3 ± 4.3	27.1 ± 7.3	25.9 ± 5.1	0.98	0.38	0.47	0.03	[24.1; 27.71]
Time (minutes)	24.1 ± 8.3	26.4 ± 9.1	29.1 ± 10.1	0.02	0.97	0.38	0.04	[23.88; 29.64]
	**Downwind**							
Mean velocity (knots)	23.5 ± 2.3 ^B^	22.1 ± 1.9	19.3 ± 3.1	2.2	0.12	0.0	0.335	[22.6; 22.48]
VMG (knots)	18.7 ± 2.6 ^B^	17.1 ± 2.1^C^	14.1 ± 3.1	0.27	0.76	0.0	0.356	[15.45; 17.49]
Distance (km)	10.6 ± 4.9	11.1 ± 4.7	10.4 ± 4.1	1.7	0.19	0.91	0.004	[9.3; 12.07]
Maneuvers (number)	23 ± 5.1	22.8 ± 2.2	24.6 ± 5.8	1.01	0.37	0.66	0.02	[21.98; 24.82]
Time (minutes)	14.2 ± 6.5	15.6 ± 5.4	17.5 ± 6.7	0.12	0.88	0.39	0.046	[13.98; 17.87]
	**Beam reach**							
Mean velocity (knots)	24.9 ± 1.5 ^B^	24.3 ± 1.3 ^C^	21.5 ± 2.3	0.97	0.38	0.0	0.728	[22.77; 24.22]
VMG (knots)	24.6 ± 1.5 ^B^	24.1 ± 1.4 ^C^	21.3 ± 2.3	0.69	0.5	0.0	0.409	[22.54; 23.99]
Distance (km)	2.6 ± 0.2	2.6 ± 0.2	2.7 ± 0.1	0.85	0.43	0.45	0.039	[2.57; 2.69]
Maneuvers (number)	0	0	0	2.3	0.11	0.61	0.024	[−0.07; 0.35]
Time (minutes)	2.7 ± 0.4 ^B^	3 ± 0.3 ^C^	3.8 ± 0.7	5.76	0.006	0.0	0.445	[3.01; 3.46]

^B^: statistically significant difference between T1 and T3; ^C^: statistically significant difference between T2 and T3. T1: high-level sailors; T2: medium-level sailors; T3: low-level sailors; CI: Confidence Interval; *d*: Effect Size.

**Table 3:** Maximum and minimum values of the analyzed variables of sailors with different performance levels.

Variable	Upwind			Downwind			Beam Reach		
	T1	T2	T3	T1	T2	T3	T1	T2	T3
Mean velocity (knots)	17.3–21.4	16.6–19.6	13.4–19.6	21.4–27.9	19.6–25.6	14.6–24.3	22.4–27.6	22.2–26.9	17.1–25.6
VMG (knots)	9.7–13.9	8.6–13.9	6.2–12.7	15.7–23.6	14.7–20.4	8.2–19.9	22.1–27.2	21.4–26.5	17.9–25.5
Distance (km)	10.3–22.7	10.3–22.1	10.8–23.8	6.9–17.8	7.3–18.4	7.6–19.3	2.1–2.8	2.3–2.8	2.3–2.8
Maneuvers (number)	18–31	18–45	19–37	17–37	19–26	17–42	0	0–4	0–2
Time (minutes)	16–38	19–46	17–55	9–29	11–24	11–35	2–3	2–4	3–5

T1: high-level sailors; T2: medium-level sailors; T3: low-level sailors.

## Data Availability

Publicly available datasets were analyzed in this study. https://site-isaf.soticcloud.net/sailors/sailor_search.php?includeref=18856&sailorid=&sailorsurname=/https://www.sapsailing.com/gwt/Home.html#StartPlace:null.
